# Peer Review of “No Time to Waste: Real-World Repurposing of Generic Drugs as a Multifaceted Strategy Against COVID-19”

**DOI:** 10.2196/24481

**Published:** 2020-09-30

**Authors:** Susan Howlett

**Affiliations:** 1 Department of Pharmacology Dalhousie University Halifax, NS Canada

**Keywords:** COVID-19, drug repurposing

*This is a peer review submitted for the paper “No Time to Waste: Real-World Repurposing of Generic Drugs as a Multifaceted Strategy Against COVID-19.”*

## Round 1 Review

### General Comments

Drug repurposing offers the chance to quickly move potentially effective drugs forward to treat COVID-19. The authors note that drug candidates should ideally have a track record of safety, affordability, and accessibility. The authors use the literature to support the use of four classes of generic drugs that target the pathophysiology of COVID-19: (1) the histamine H2 receptor antagonists cimetidine and famotidine; (2) the antiplatelet agent/phosphodiesterase inhibitor dipyridamole; (3) the phosphodiesterase-5 inhibitor sildenafil; and (4) the cholesterol-lowering agents fenofibrate and bezafibrate. These drugs are affordable and supported by decades of safety data. They also argue that this approach can be used to identify other drugs to address this urgent clinical need. The work is provocative and addresses a topic of great importance. Although it is generally well written, there are areas where this manuscript could be revised to improve clarity and readability, as outlined below.

### Specific Comments

#### Major Comments

##### Overall

The authors use many nonstandard abbreviations. Also, many of these are used only once or a few times. To improve readability, especially for this review article, please greatly reduce the number of abbreviations, especially nonstandard ones that are hardly used. Also, please make sure that all of your abbreviations are defined.

There are a few new papers in this rapidly evolving area that the authors may wish to include in this review. These are (1) cimetidine and famotidine: an ongoing RCT for famotidine (NCT04370262; https://pubmed.ncbi.nlm.nih.gov/32446698/); (2) dipyridamole: an observational study (NCT04424901); (3) sildenafil citrate: not yet recruiting (NCT04489446).

##### Abstract

The abstract is a bit vague about the nature of the drugs used and the pathophysiology targeted. It would be important to mention the drug classes as well as (or instead of) the specific drugs of interest in the abstract. In addition, it would be helpful if the authors could mention some of the critical underlying pathophysiological mechanisms that these drugs target with respect to COVID-19 (eg, anti-inflammatory, antiviral, cardioprotective, etc).

##### Background section

As for the abstract, please refer to the drug classes as well as the specific drugs of interest.

##### Pathophysiology of COVID-19 section

In this section, it is not clear whether the authors mean inpatients or all affected individuals. In Table 1, it is easy to see fever in 79%-98% of all affected individuals, but it is more difficult to understand the 59% with sepsis refers to individuals in the community. Is this at presentation? These numbers need a better context. It would also be helpful to have references in the table.

Rearranging Table 1 would improve readability.

System, Clinical Finding, Prevalence, Reference

Respiratory Fever 79%-98% X

Cough 58%-79% X

etc…

Also, in this section, the authors refer to elevation of various plasma inflammatory biomarkers in COVID-19. Please list these, or the major ones. Alternatively, please clarify if they differ from what is listed in Table 1.

##### Potential Therapies Within the Current Pharmacopoeia Section

On page 4, in the sentence starting with “All three drugs…,” the authors argue the benefits of strategies that utilize the safest drugs with pleiotropic effects to treat COVID-19. This is an interesting and important point. However, this sentence is too long to be easily understood. Please revise and expand to clarify meaning.

Table 2: This table would be improved by the addition of specific references.

##### Dipyridamole section

Page 3, top paragraph: The sentence starting with “Within the 200-400 mg…” needs a reference.

Page 4, second paragraph: For the sentence starting with “Examples include…,” please discuss/explain prednisolone and explain what is meant by “widening of the therapeutic window of glucocorticoid activity.”

##### Sildenafil section

The authors mention the ongoing phase 3 trial of sildenafil (100 mg daily for 14 days in patients with COVID-19 and give the trial number [NCT04304313]). It would be helpful to include trial numbers in Table 2.

##### Conclusions:

The sentence beginning with “The efforts we make now to facilitate…” is really long and complex. It is hard to follow as written. Please clarify.

#### Minor Comments

Please bold headings for Sildenafil and Fenofibrate / Bezafibrate for consistency.

Please be consistent with SARS-CoV—the V is not capitalized in all cases.

Page numbers are not sequential.

